# Mechanism and clinical value of exosomes and exosomal contents in regulating solid tumor radiosensitivity

**DOI:** 10.1186/s12967-022-03392-w

**Published:** 2022-04-28

**Authors:** Huihui Sun, Rui Sun, Xing Song, Wendong Gu, Yingjie Shao

**Affiliations:** Department of Radiation Oncology, The Third Affiliated Hospital of Soochow University, 185 Juqian Street, Changzhou, 213003 China

**Keywords:** Exosomes, Radiosensitivity, microRNA, Tumor

## Abstract

Radiotherapy is among the routine treatment options for malignant tumors. And it damages DNA and other cellular organelles in target cells by using ionizing radiation produced by various rays, killing the cells. In recent years, multiple studies have demonstrated that exosomes are mechanistically involved in regulating tumor formation, development, invasion and metastasis, and immune evasion. The latest research shows that radiation can affect the abundance and composition of exosomes as well as cell-to-cell communication. In the environment, exosome-carried miRNAs, circRNA, mRNA, and proteins are differentially expressed in cancer cells, while these molecules play a role in numerous biological processes, including the regulation of oncogene expression, mediation of signaling pathways in cancer cells, remodeling of tumor-related fibroblasts, regulation of cell radiosensitivity, and so forth. Therefore, elucidation of the mechanism underlying the role of exosomes in radiotherapy of malignant tumors is crucial for improving the efficacy of radiotherapy. This review will summarize the research advances in radiosensitivity of malignant tumors related to exosomes.

## Introduction

The morbidity and mortality of cancer have been increasing rapidly during the past half century. In 2020, approximately 19.3 million new cancer cases and almost 10 million cancer deaths were reported worldwide [1]. At present, there has been a rapid progress in early diagnosis techniques for cancer (nano-flow detection technology [2] and circulating tumor DNA [3] ) as well as new therapeutic means (immunotherapy [4] and targeted therapy [5]. However, cancer remains a significant public issue threatening the human health. In recent years, biological effects of exosomes on cancer formation, development and prognosis have gradually been unveiled, while the application of exosomes as a biomarker for disease diagnosis and prognostic prediction or a drug delivery vehicle has rapidly been promoted [6–8]. In 1987, Johnstone et al. [9] observed an exocytotic pleomorphic vesicular body termed exosome during the study of maturation of sheep reticulocytes. In 1996, Raposo et al. [10] showed that exosomes play a role in immune system by acting as a vehicle for intercellular communication, thus entering people’s vision. Later on, Ekström et al. [11] revealed that genetic materials can be delivered via exosomes in certain cells in order to reciprocally regulate gene expression by protein and miRNA. This important finding suggests to us exosomes may be closely related to human physio-pathological state and potentially serve as a novel therapeutic target for diseases.

**Fig. 1:**
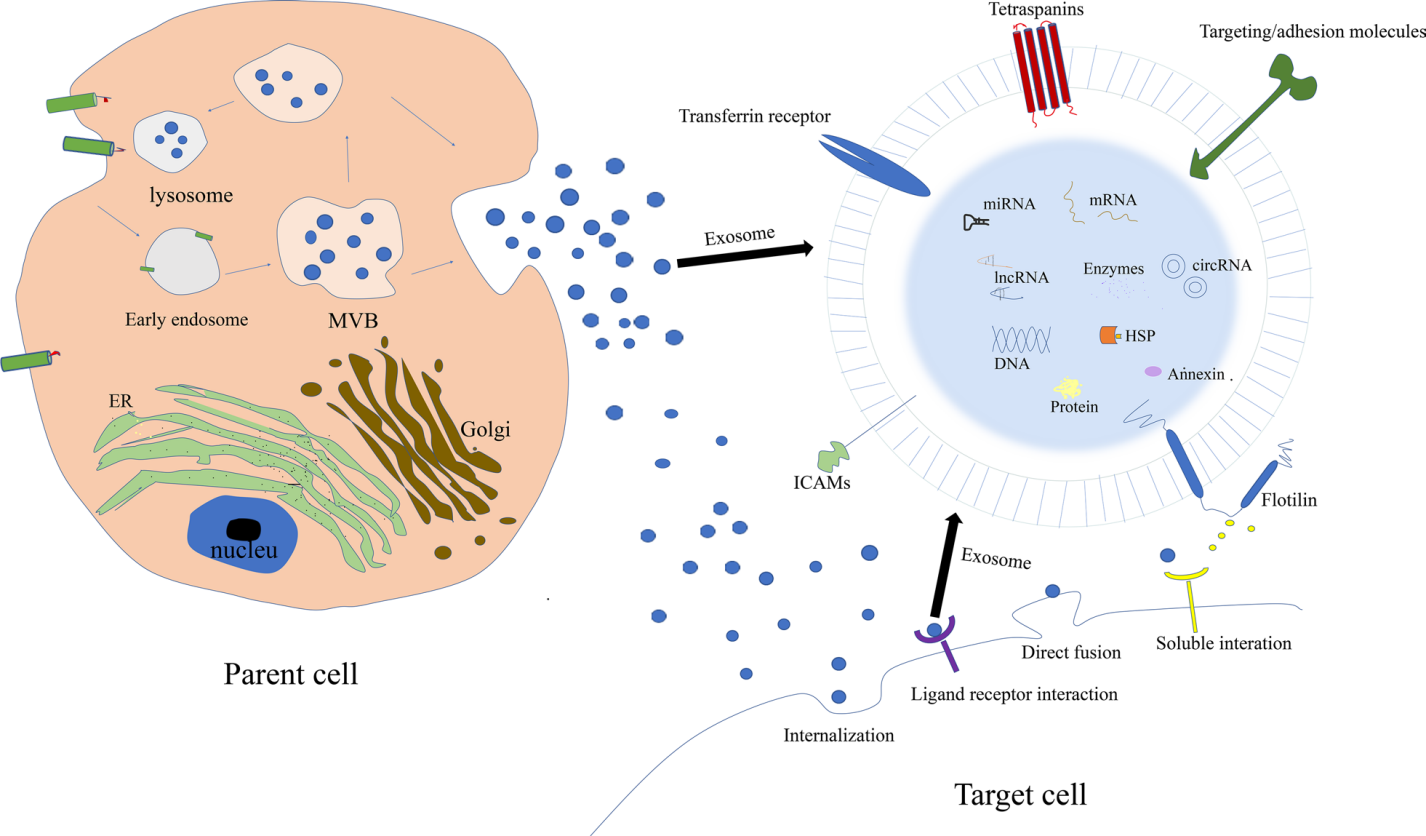
Exosomes biogenesis and release mechanism to recipient cells

Exosomes are a class of bilayer-bound extracellular vesicles with a diameter of 30–100 nm, which contain various bioactive components such as protein, DNA, mRNA, miRNA, other non-coding RNA, lipid, and so forth. And they have been widely detected in body fluids, including cerebrospinal fluid, saliva, breast milk, blood and urine (among others) [12–15]. It is generally recognized that exosomes are derived from early endosomes formed by cell membrane invagination. Early endosomes bud inward and selectively encapsulate protein, nucleic acids, lipid, and others to form multivesicular bodies. While a part of multivesicular bodies fuse with intracellular lysosomes to degrade the contents, the others fuse with plasma membrane under the traction of intracellular molecular motor to secret exosomes for exerting biological function [16]. Upon release, exosomes act as a new vehicle for cell-to-cell communication to play an important role in various biological processes, including intercellular signal transduction, substance delivery, gene expression, and biochemical metabolism. Exosomes may directly bind to target cell receptor via their surface ligands to stimulate target cells, or function in horizontal transfer of bioactive components into target cells through fusing with target cells and endocytosis, thus affecting cell function (Fig. 1).

**Fig. 2:**
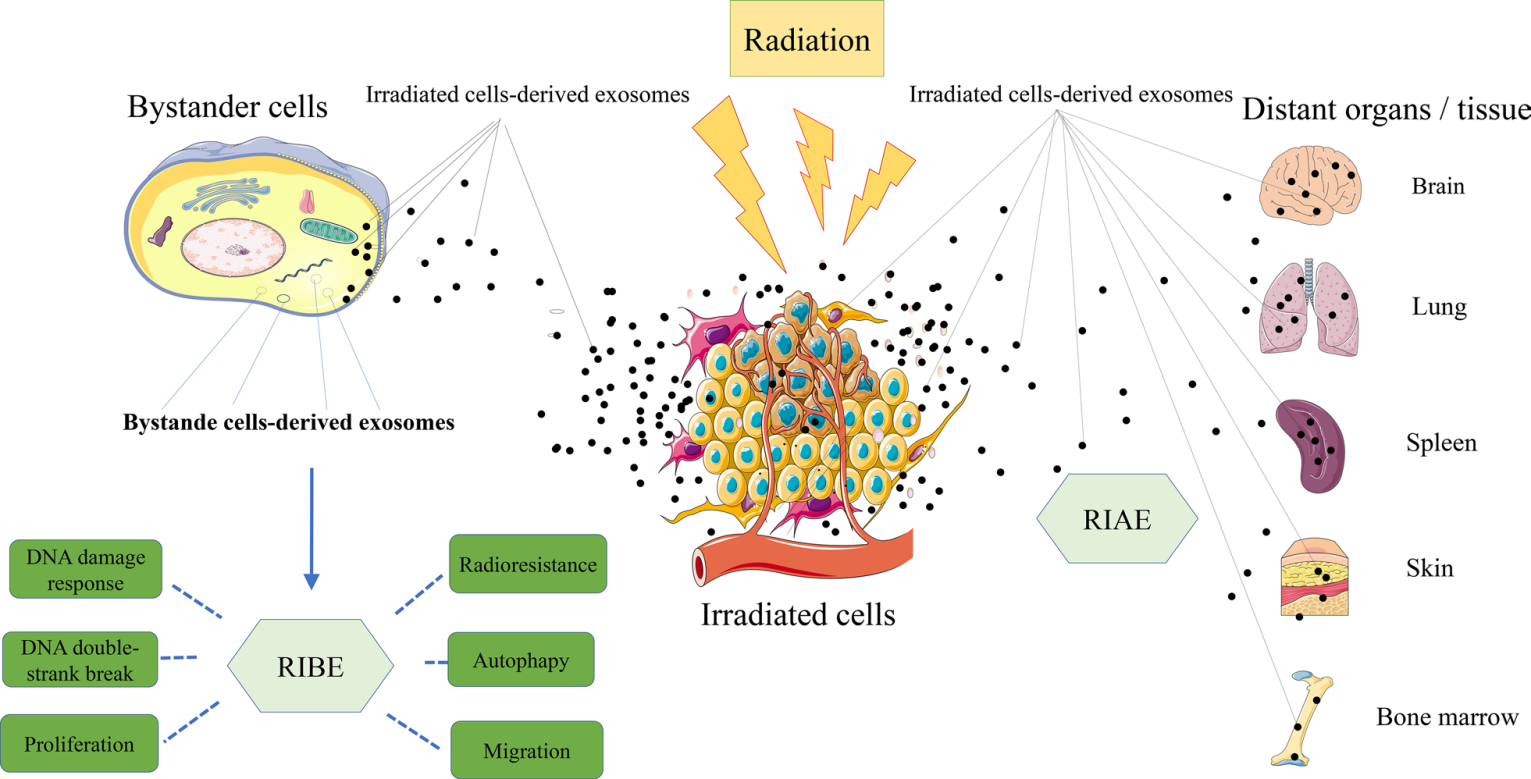
Exosomes and exosomal contents participate in multiple RIBE, including DNA damage response, DNA double-strank break, cell proliferation and migration changes, promoted cell autophagy, and to tumor cell radioresistance changes. The contents of exosomes are packaged and protected to escape degradation, cross various physiological barriers, target unirradiated distant tissues and cells, and induce RIAE

**Fig. 3:**
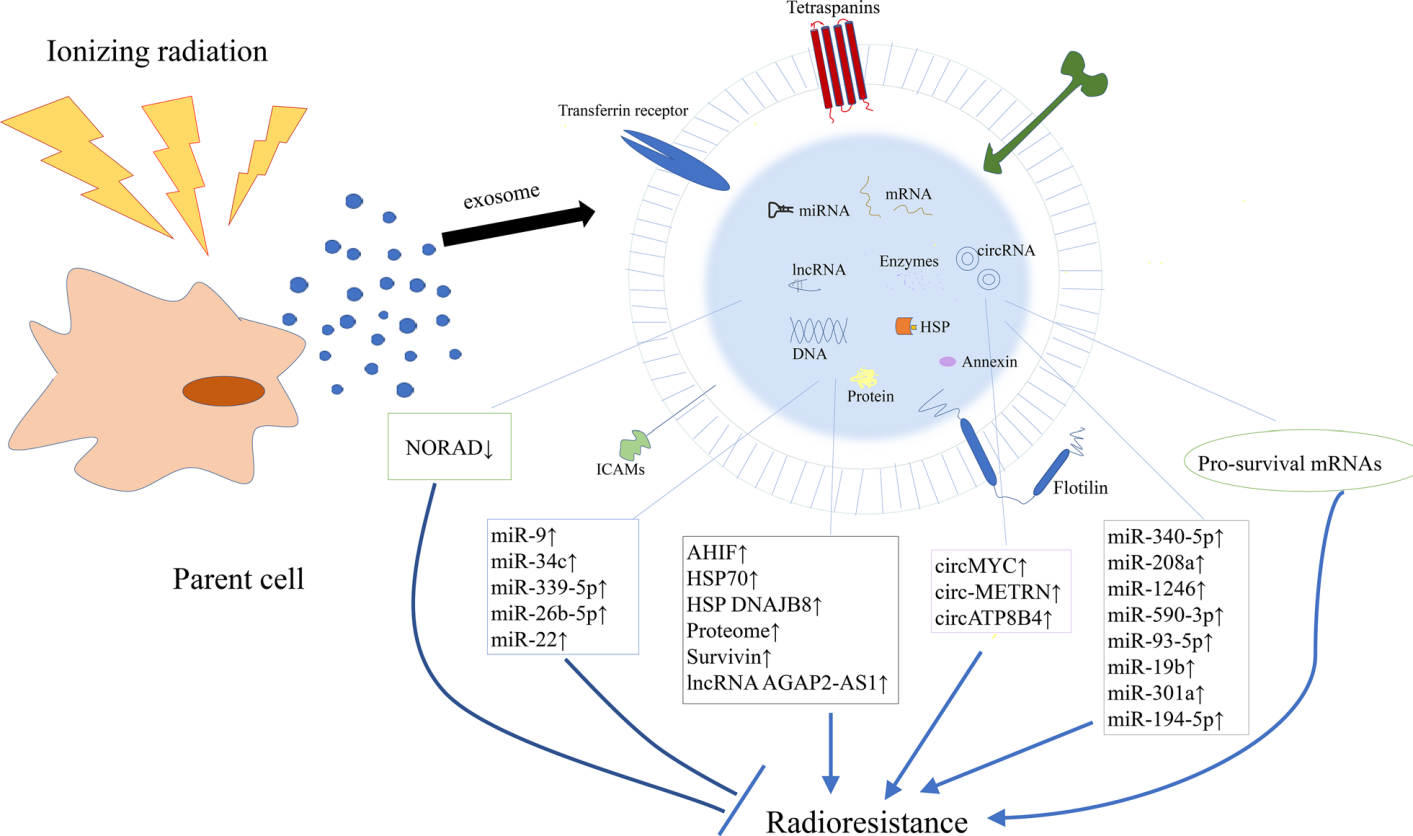
An overview of exosomes/exosomal cargo contents in the regulation of cancer radiosensitivity. Exosomes/exosomal cargo contents exert important function to regulate the radiosensitivity of cancer cells, via complicate interaction with multiple biological processes including DNA damage response, cell cycle and apoptosis, hypoxic tumor microenvironment, epithelial–mesenchymal transition, cancer stem cells and radiation-induced signaling pathways

**Fig. 4:**
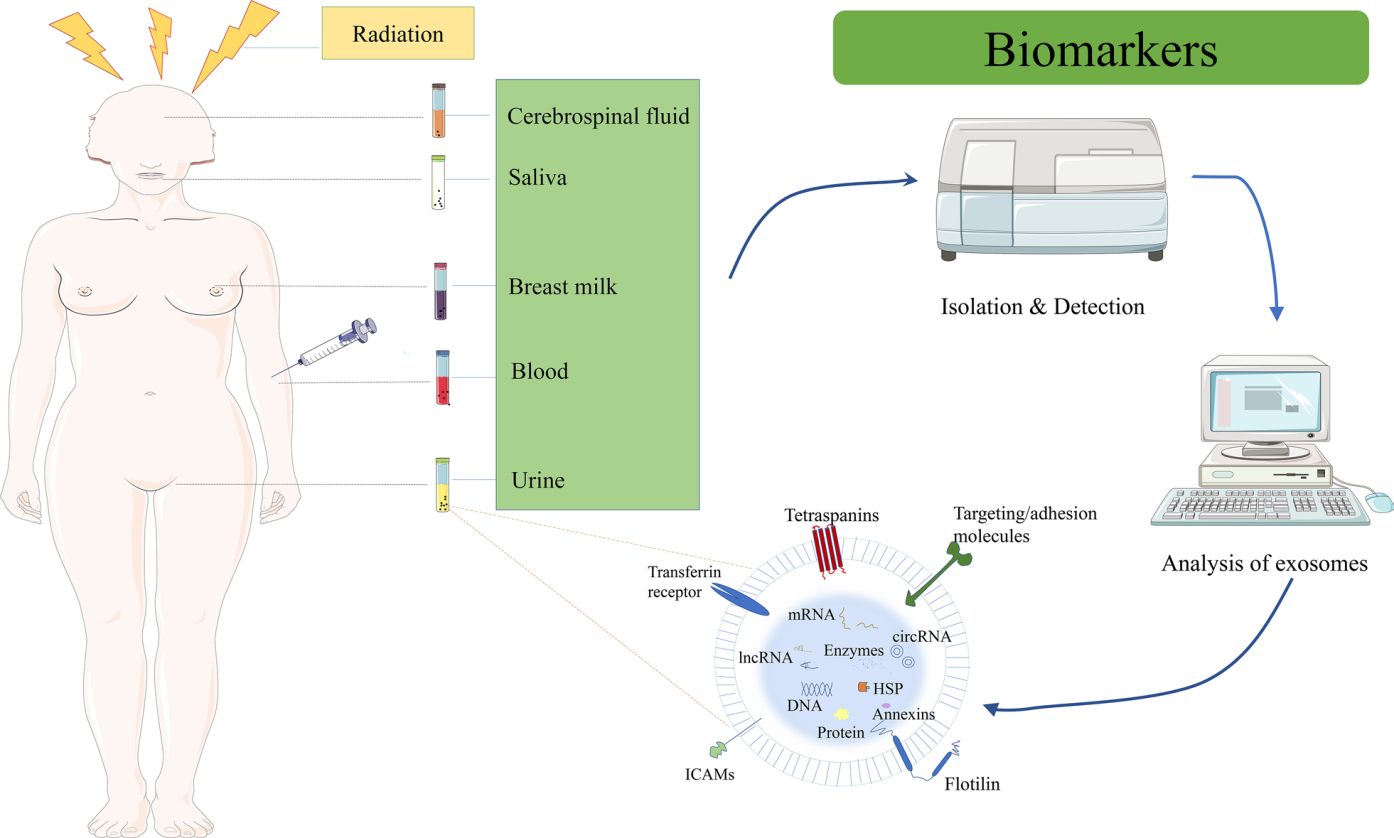
Exosomes can be isolated from body fluids, including cerebrospinal fluid, saliva, breast milk, blood, and urine (among others). Analysis of the contents of exosomes exposed to radiation, with altered levels of exosome content expression, which makes exosomes promising as a biomarker for early diagnosis and prognostic prediction of diseases

With the deepening research on exosome function, the mechanism underlying the role of exosomes in tumorigenesis has been further unveiled. The above achievement provides a theoretical basis for implementing cancer precision therapy and individualized therapy. It has been shown that while tumor cells secret much higher number of exosomes than normal cells, the level of exosomes is correlated with cancer prognosis [17]. Moreover, exosomes carry source cell-specific bioactive components including protein, mRNAs and miRNAs, which could indicate an alteration in genetics or signaling within cancer cells. Therefore, exosomes may have bright prospects in serving as a biomarker for early diagnosis and prognostic prediction of cancer [18, 19]. While good biocompatibility, low immunogenicity, liposolubility and nano-scale diameter allow exosomes to penetrate various biological barriers such as blood brain barrier, the bilayer membrane structure of exosomes can function as a natural protective barrier for protecting their contents from hydrolysis by the surrounding enzymes. Hence, exosomes could be used as a delivery vehicle in gene therapy or an ideal drug carrier [20–23]. Radiotherapy is a routine treatment option for malignant tumors. And it damages DNA and other cellular organelles in target cells by using ionizing radiation produced by various rays, killing the cells. However, while radiotherapy kills tumor cells, it can also cause damage to bystander cells and even distant tissues and organs, which is called radiation-induced bystander effects (RIBE) and radiation-induced abscopal effects (RIAE) (Fig. 2) [24, 25]. There is an increasing number of evidences that exosomes play a significant role in RIBE and RIAE, including activating the RIBE and inducing biological effects such as genomic instability, stress response, apoptosis and changes in cell proliferation after radiotherapy (Fig. 2) [26–28]. It has recently been shown that radiation leads to alterations in the secretory rate and components of released exosomes in both malignant tumor cells and normal cells [27] , which are essential for radioresistance of radiated cells as well as signal transmission between radiated cells and non-radiated cells. Not only that, exposure to radiation also increases the uptake of exosomes by cells (Fig. 2). Studies have found that radiation can elicit information communication between cancer cells and tumor microenvironment. Exosomes can be used as a means of intercellular communication. In the environment, exosome-carried miRNAs, circRNA, mRNA, and proteins are differentially expressed in cancer cells, while these molecules play a role in numerous biological processes, including the regulation of oncogene expression, mediation of signaling pathways in cancer cells, remodeling of tumor-related fibroblasts, regulation of cell radiosensitivity, and so forth [29–32]. Hence, elucidation of the mechanism underlying the role of exosomes in radioresponse is crucial for improving the efficacy of radiotherapy (Figs. 3, 4, Tables 1, 2).

**Table 1:** Roles of exosomes/exosomal cargo contents in regulating cancer radiosensitivity

Exosomes/Exosomal contents	Cancer types	Mechanism	Function	References
Exosomes (↑)	HNSCC	Increase DNA double-strand break repair and promote proliferation	Promote radioresistance	[34]
Exosome miR-9 (↑)	HPV + HNSCC	Polarize macrophages into M1 phenotype via downregulating PPARγ	Promote radiosensitivity	[35]
Exosome miR-34c(↑)	NPC	Block the EMT process by directly targeting β-catenin	Promote radiosensitivity	[37]
Exosome circMYC (↑)	NPC	Sponge miR-20b-5p and let-7e-3p	Promote radioresistance	[38]
LMP1-positive exosomes (↑)	NPC	Stimulate p38 MAPK signaling through the exosomal transfer of LMP1 to recipient cells	Promote radioresistance	[30]
Exosome miR-340-5P(↑)	ESCC	Alleviate radiation-induced apoptosis and accelerate DNA damage repair by directly targeting KLF10	Promote radioresistance	[29]
Exosome miR-339-5p(↑)	ESCC	Directly target Cdc25A	Promote radiosensitivity	[46]
Exosome NORAD(↓)	ESCC	Promote exosomal miR-199a-5p dispersion	Promote radiosensitivity	[47]
Exosome miR-208a(↑)	NSCLC	Target p21 with a corresponding activation of the AKT/mTOR pathway	Promote radioresistance	[48]
Exosome miR-1246(↑)	NSCLC	Target the DR5	Promote radioresistance	[49]
Exosome miR-26b-5p(↑)	LUAD	Target ATF2 in DNA damage	Promote radiosensitivity	[53]
Exosome lncRNA AGAP2-AS1(↑)	NSCLC	Downregulate miR-296 and upregulate NOTCH2	Promote radioresistance	[54]
Exosome HSP70(↑)	NSCLC	Synthesize hypoxia-related genes	Promote radioresistance	[55]
Exosomes(↑)	CRC	Promote colorectal cancer stem cell phenotype	Promote radioresistance	[61]
Exosome miR-590-3p(↑)	CRC	Regulate the CLCA4-dependent PI3K/Akt signaling pathway positively	Promote radioresistance	[62]
Exosome miR-93-5p(↑)	CRC	Downregulate FOXA1 and upregulate TGFB3	Promote radioresistance	[63]
Exosome miR-19b(↑)	CRC	Target FBXWT to promote colorectal cancer stem cell stemness	Promote radioresistance	[64]
Exosome circ-0067835(↓)	CRC	Upregulate miR-296-5p and downregulate IGFIR	Promote radiosensitivity	[31]
Exosome AHIF (↑)	Glioblastoma	AHIF-mediated p53 downregulation and anti-apoptosis	Promote radioresistance	[77]
Exosome miR-301a (↑)	Glioblastoma	Directly target TCEAL7, TCEAL7 negatively regulate the Wnt/β-catenin pathway	Promote radioresistance	[78]
Exosome circ-METRN (↑)	Glioblastoma	MiR-4709-3p/GRB14/PDGFRα pathway	Promote radioresistance	[82]
Exosomes (↑)	Glioblastoma	Increase oncogenic miRNAs、mRNAs and pro-survival proteasome pathway;decrease levels of tumor-suppressive miRNAs and mRNAs	Promote radioresistance	[83]
Exosome circATP8B4(↑)	Glioblastoma	MiR766 sponge	Promote radioresistance	[84]
Exosomes (↑)	Neuroblastoma	Activate downstream dependent survival pathway	Promote radioresistance	[86]
Exosome miR-194-5p (↑)	Pancreatic cancer	Enhance DNA damage response in TRCs	Promote radioresistance	[87]
Exosomes (↑)	Prostate cancer	Induce autophagy	Promote radiosensitivity	[89, 90]
Exosome HSP DNAJB8(↑)	Renal cell carcinoma	Maintain RCC CSCs/CICs	Promote radioresistance	[92, 93]
Exosome survivin(↑)	cervical carcinoma	Bystander effect	Promote radioresistance	[95]
Exosome miR-22(↑)	cervical carcinoma	Upregulate apoptotic pathway	Promote radiosensitivity	[96]
Exosomes (↑)	Melanoma	Stimulate tumor cell death	Promote radiosensitivity	[97]
Exosomes (↑)	Breast cancer	Induce autophagy	Promote radiosensitivity	[89, 90]
Exosomes (↑)	Breast cancer	Paracrine and juxtacrine signaling	Promote radiosensitivity	[98]
Exosomes proteome (↑)	Breast cancer	Hypoxic microenvironments; upregulate pro-survival factors	Promote radioresistance	[99]
Exosomes (↑)	Breast cancer	Increase the activity of exosomal secretory pathway	Promote radioresistance	[79]

HNSCC: Head and neck squamous cell cancer; NPC: Nasopharyngeal carcinoma; ESCC: Esophageal squamous carcinoma; NSCLC: Non—small cell lung cancer; LUAD: Lung adenocarcinoma; CRC: Colorectal cancer

**Table 2:** Exosomes as diagnostic, prognostic or predictive biomarker in solid tumor

Exosomes/Exosomal contents	Cancer types	Specimen origin	Biomarker function	References
Exosome circMYC	NPC	Serum	Differentiate radioresistant patients from patients with radiosensitive NPC	[38]
Exosome miR-339-5p	ESCC	Serum	Predict radiotherapeutic response	[46]
Exosome miR-96	NSCLC	Plasma	Diagnostic and prognostic marker of radioresistant NSCLC	[50]
Exosome miR-378	NSCLC	Serum	Predict radiotherapeutic response	[51]
Exosome miR-29a-3p miR-150-5p	NSCLC	Serum	Predict radiotherapeutic response; radiation-related	[52]
Exosome HSP70	NSCLC	Plasma	Predict radiotherapeutic response	[55]
Exosome miR-663a	CRC	Plasma	Predict radiotherapeutic response	[67]
Exosome miR-574-3p	Glioblastoma	Serum	Predict radiotherapeutic response	[85]
Exosome miR-379-5p miR-654-3p	Prostate cancer	Serum	Predict radiotherapeutic response	[91]
Exosome proteome	Breast cancer	Body fluids	Differentiate radiation-resistant tumors	[99]

### Head and neck squamous cell carcinoma (HNSCC)

Over 95% of head and neck cancers are histopathologically classified as squamous epithelial cell carcinoma. At present, the tumor location in patients with HNSCC can be precisely detected using imaging technique, and better radiotherapy strategies have been developed. However, while not all patients with HNSCC exhibit the sensitivity to radiotherapy, a portion of the patients are resistant to the radiotherapy [33]. It has been shown that the exposure of HNSCC cells BHY and FaDu to γ-ray leads to an increased secretion of exosomes. Moreover, co-culture of BHY cells with the exosomes conferred the cells with radioresistance and promoted the proliferation of recipient cells within 6 h after administration with exosomes. In this case, the exosomes facilitated the radioresistance through promoting DNA double-strand break repair [34]. Meanwhile, studies have revealed that miR-9 enriched exosomes isolated from human papilloma virus (HPV) positive HNSCC patients can transform macrophages into M1 phenotype and facilitate the radiosensitivity of these patients [35]. Nasopharyngeal carcinoma (NPC) is a subtype of head and neck cancer frequently occurring in Southeast Asian countries and Southern China [36]. Wan et al. [37] reported that miR-34c-overexpressing exosomes inhibit malignant behaviors of NPC and restore its radioresistance. Exosomal miR-34c down-regulates epithelial-mesenchymal transformation in NPC by inhibiting β-catenin, enhancing the radiosensitivity. Luo et al. [38] collected and analyzed circulating exosomal circMYC samples from 210 NPC patients, and found that while circulating exosomal circMYC is highly expressed in the patients, the overexpression of circMYC promotes NPC cell proliferation as well as the radioresistance. It has been demonstrated that intercellular transport of LMP1 can occur via extracellular vesicles or exosomes, and exosome-mediated intercellular transport is closely related to the role of Epstein‐Barr virus(EBV) in carcinogenesis [39]. In addition, Zhang et al. [30] provided evidence that LMP1-containing exosomes derived from NPC cell line CNE1 can activate P38 MAPK signaling pathway, conferring radioresistance to recipient NPC cells. This finding confirms from a lateral perspective that a small portion of LMP1-expressing cells enhance the radioresistance of NPC cells presumably by affecting the infected host and regulating tumor microenvironment.

### Esophageal cancer (EC)

EC is among malignant tumors seriously endangering people’s health and life. Radiotherapy has been a main treatment option for EC, particularly for cervical and upper thoracic EC with a relatively big surgical difficulty. Notably, EC patients vary greatly in the efficacy and prognosis of radiotherapy, suggesting a significant difference in sensitivity of esophageal cancer cells to radiotherapy among the individuals [40]. Hence, how to improve sensitivity of EC cells to radiotherapy has become a pivotal issue in the field of radiation oncology. Multiple studies have shown that exosomes derived from radiation exposed cells in the microenvironment facilitate the efficacy of radiotherapy possibly via the bystander effect and distal effect [41–44]. Hypoxia is a main reason to cause radioresistance. In this case, hypoxic tumor cells may release a certain type of exosome which are subsequently engulfed by the neighboring cells in the tumor environment, eliciting a series of biological changes [45]. In accordance with the above theory, Chen et al. [29] found that exosomes derived from hypoxic esophageal squamous cell carcinoma (ESCC) cells down-regulate the radiosensitivity of recipient cells through transporting miR-340-5p. Furthermore, miR-340-5p negatively regulates KLF10 and UVRAG, inhibiting ionizing radiation-induced apoptosis and facilitating DNA damage repair. Notably, Luo et al. [46] found that while miR-339-5p can be selectively secreted into blood via exosomes, the relatively high level of serum miR-339-5p is positively correlated with the radiosensitivity and survival rate. Moreover, exosome-derived miR-339-5p can mediate the radiosensitivity of ESCC by down-regulating Cdc25A and predict pathological response of locally advanced ESCC to pre-operative radiotherapy, indicating that the miR-339-5p may become a promising non-invasive biomarker. Besides, a high expression of lncRNA NORAD has been identified as an indicator of radioresistance for ESCC. While NORAD can be activated by radiation via enhanced enrichment of H3K4me2, highly expressed NORAD competitively binds to PUM1, decreasing the processing of pri-miR-199a1 and down-regulating the expression of miR-199a-5p. Knocking out NORAD in EC cells led to up-regulation of miR-199a-5p-targeted EEPD1 as well as down-regulation of ATR/Chk1 signaling in exosomes, thereby enhancing radiosensitivity of esophageal cancer cells [47]. 

### Non-small cell lung cancer (NSCLC)

Radiotherapy or combined chemoradiotherapy is currently the main treatment option for patients with intermediate and advanced-stage NSCLC who lost the opportunity of radical surgery. Numerous studies have demonstrated that miRNA-containing exosomes act as a carrier for regulating radiosensitivity of tumor cells as well as a tool for monitoring the efficacy of radiotherapy. Tang et al. [48] reported that an alteration in serum miRNA expression pattern occurs in NSCLC patients after radiotherapy, including a significant increase in the level of serum miR-208a. Serum miR-208a-containing exosomes can be taken up by NSCLC cells, and miR-208a subsequently promotes the proliferation of the cancer cells and inhibits apoptosis through targeting p21 and AKT/mTOR pathway, thus affecting the radioresistance of cells. Yuan et al. [49] found that exosomes derived from irradiated tumor cells promote the radioresistance of recipient cells by transporting miR-1246. Zheng et al. [50] observed that the level of plasma miR-1246 and miR-96 is significantly increased in NSCLC patients compared with normal individuals, while the level of exosomal miR-96 in patients with radioresistant NSCLC is markedly higher than that in those with radiosensitive NSCLC. They, therefore, proposed that exosomal miR-96 could serve as a noninvasive diagnostic and prognostic biomarker for radioresistant NSCLC. In the meantime, Zhang et al. [51] showed that the level of serum exosomal miR-378 is markedly reduced in over 50% of NSCLC patients following radiotherapy, implying that miR-378 may act as an indicator for response of NSCLC patients to radiotherapy. It has also been demonstrated that among 752 exosomal miRNAs identified from patients with locally advanced NSCLC, the expression of miR29a-3p and miR150-5p is decreased with the increasing radiation dose [52]. Besides, exosomal miRNAs derived from tumor or stromal cells have been shown to play a role in the progression and therapy resistance of lung adenocarcinoma. Han et al. [53] proved that exosomal miR-26b-5p facilitates radiosensitivity of lung adenocarcinoma by regulating ATF2. Meanwhile, Zhang et al. [54] confirmed that exosomal lncRNA AGAP2-AS1 derived from M2 macrophages can enhance radioresistance of radiation-resistant NSCLC through down-regulating microRNA-296 and up-regulating NOTCH2. Ostheimer et al. [55] presented data showing that exosomal Hsp70 significantly enhances radioresistance of NSCLC.

### Colorectal cancer (CRC)

CRC remains one of digestive tumors with the highest incidence. And radiotherapy is an important part of the comprehensive treatment of CRC. Previous studies have shown that the tumor microenvironment mediates the resistance to radiotherapy [56]. As an important component of the tumor environment, tumor-related fibroblasts are significantly involved in the resistance to radiotherapy [57–60]. Liu et al. [61] found that exosomes derived from tumor-related fibroblasts increase the stemness of CRC cells by activating TGF-β signaling pathway, enhancing radioresistance of the cancer cells. It has also been demonstrated that exosomal miR-590-3p derived from tumor-related fibroblasts enhances the radioresistance of CRC cells by regulating CLCA4-dependent PI3K/Akt signaling pathway in a positive feedback loop [62]. Likewise, Chen et al. [63] observed that tumor-related fibroblast derived exosomes for transporting miR-93-5p promote the proliferation and colony formation of tumor cells and inhibit apoptosis through down-regulating FOXA1 and up-regulating TGFB3, thereby enhancing the radioresistance of CRC cells. In the meantime, Sun et al. [64] proved that exosomal miR-19b derived from CRC cells inhibits the maintenance of CRC stem cells and enhances radiosensitivity of the cancer cells by activating Wnt/β-catenin signaling pathway via down-regulation of FBXW7. Besides, circRNA has been demonstrated to regulate radiosensitivity of CRC cells through exosome-mediated transport [65, 66]. Studies have revealed that serum exosomal circ_0067835 is remarkably upregulated in CRC patients following radiotherapy, while being transported via exosomes in the patients. Notably, a combined functional assay demonstrated that knocking out circ_0067835 regulates the expression of IGF1R by sponging miR-1236-3p, thus suppressing the progression of CRC and enhancing radiosensitivity of the cancer [31]. Moreover, Bjørnetrø et al. [67] showed that miR-663a is expressed in exosomes released from hypoxic CRC cells and provided evidence that exosomal miR-663a is related to radioresistance caused by hypoxic tumor environment.

### Glioblastoma

Glioblastoma is the most common primary central nervous system malignant tumor [68]. While surgery is the main treatment option for glioblastoma, unsuccessful surgical resection often occurs mainly due to the fact that glioblastoma usually displays an invasive growth invading parenchyma [69, 70]. Hence, radiotherapy has become a main adjuvant therapy for glioblastoma patients [71]. However, radiation tolerance even radioresistance could significantly affect the treatment and prognosis of glioblastoma patients receiving radiotherapy because of the presence of individual variation and tumor heterogeneity [72–74]. It has been shown that radiation exposure can lead to a significant release of CTGF-containing exosomes, being involved in the tumor formation. Co-culture of CTGF-containing exosomes with unirradiated cells may cause the overexpression of CTGF in recipient cells. Moreover, radiation has been found to affect the abundance of exosomes; in particular, it may change the components of exosomes and facilitate the migratory phenotype during the uptake process [75]. Kucharzewska [76] found that exosomes reflect the hypoxic status of glioma cells and mediate hypoxia-dependent activation of vascular cells during tumor development. Thereafter, Dai et al. [77] validated that AHIF is markedly up-regulated in cancerous tissue and radiation resistant glioblastoma, while AHIF promotes the progression and radioresistance of glioblastoma via exosomes. The above findings suggest that AHIF may serve as a biomarker for predicting the progression and radioresistance of glioblastoma, while exosomes could potentially become a therapeutic target for glioblastoma. Yue et al. [78] showed that exosomal miR-301a released from hypoxic glioblastoma cells activates Wnt/β-catenin signaling pathway through inhibiting the expression of tumor suppressor gene TCEAL7, ultimately facilitating the tumor radioresistance. Thus, exo-miR-301a/TCEAL7-signaling axis would most likely provide new ideas for overcoming radioresistance in glioblastoma patients. In the meantime, numerous studies have demonstrated that irradiation can not only promote the generation and transport of exosomes, but also facilitate the absorption of exosomes by other cells [79–81]. Wang et al. [82] provided the first demonstration that while circ-METRN is highly enriched in low-dose radiation-induced exosomes, circ-METRN in the exosomes exerts an effect on the progression and radioresistance of glioblastoma via miR-4709-3p/GRB14/PDGFRα pathway. Meanwhile, Mrowczynski et al. [83] observed that exosomes can increase the levels of carcinogenic miRNAs (miR-889), mRNAs and survival-promoting pathway proteins, while decreasing the levels of tumor suppressive miRNAs (miR-516 and miR-365) and mRNAs, thereby promoting the survival rate of cells exposed to irradiation. Besides, Zhao et al. [84] found that circATP8B4 in RR-U251 cell derived exosomes could be transported to glioma cells in which it sponges miR-766 to enhance radioresistance of the cells. Li et al. [85] demonstrated that radiotherapy leads to a marked decrease in the level of miR-574-3p in plasma exosomes, indicating that miR-574-3p could serve as an important biomarker for monitoring the efficacy of radiotherapy.

### Other types of carcinomas

Neuroblastoma is characterized by insidious onset, high malignancy and extremely rapid progression. And radiotherapy is currently one of the breakthrough points in promoting the survival rate of neuroblastoma. Tortolici et al. [86] demonstrated that early release of the extracellular vesicles from irradiated neuroblastoma cells can induce the activation of the survival pathway, promoting the proliferation and invasion of recipient cells related to epithelial-mesenchymal transitions. Upon irradiation of these cells, EVs induce a cell cycle arrest at G2/M phase for DNA damage repair. The above process is an adaptive cellular response that is essential for the formation of a stronger phenotype of invasiveness and irradiation resistance.

Pancreatic cancer is a highly invasive malignancy. While radiotherapy plays a crucial role in the treatment of pancreatic cancer, it could lower the rate of local recurrence and increase the chance of surgical operation in locally advanced pancreatic cancer. Notably, pancreatic cancer cells are relatively insensitive to radiotherapy. Jiang et al. [87] reported that exosomes can facilitate the radioresistance through transporting proliferation-suppressing miRNAs. Strikingly, irradiation-induced dying tumor cells have been shown to regulate tumor cell repopulation. In this case, irradiated dying cells secret exosomes, induce a cell cycle arrest, and promote DNA damage repair, thus improving the survival capacity of ALDH1A1 + repopulated tumor cells.

Prostatic cancer is highly invasive, and radiotherapy is currently an effective approach for the treatment of prostatic cancer. Hurwitz et al. [88] comparatively analyzed prostatic cancer tissue specimens before and after radiotherapy and found that radiation exposure leads to a certain increase in the level of exosomal HSP72. Moreover, exosomes derived from prostatic cancer stem cells have been demonstrated to induce autophagy, while autophagy has been proved to regulate the sensitivity of tumor cells to radiotherapy [89, 90]. In addition, Yu et al. [91] showed that a number of miRNAs are detected in serum exosomes from prostatic cancer patients who underwent carbon ion radiotherapy, including miR-493-5p, miR-323a-3p, miR-411-5p, miR-494-3p, miR-379-5p, miR-654-3p, miR-409-3p, miR-543, and miR-200c-3p. Among these miRNAs, miR-379-5p and miR-654-3p could be used for predicting the sensitivity of tumor cells to carbon ion radiotherapy.

Renal cell carcinoma is among the most common malignancies in urinary system, and radiotherapy is an effective adjuvant therapy option for advanced renal cancer. It has been shown that exosomal Hsp DNAJB8, a Hsp40 family member, plays a certain role in maintaining radioresistance of CSCs/CICs in renal cell carcinoma [92, 93]. 

At present, radiotherapy is a main option for clinical treatment of intermediate and advanced cervical cancer. While a good curative effect could be achieved at the early stage of radiotherapy, the accumulating radiation dose at the later stage may lead to a decrease in sensitivity of cervical cancer cells to radiotherapy [94]. Proton beam therapy (PBT) is one type of radiotherapy, and exosomes have been recently reported to mediate PBT resistance. Proton-irradiated HeLa cervical cancer cells can secret exosomes containing a high level of survivin, facilitating tumor growth and resistance to the therapies [95]. It has been demonstrated that miR-22 enriched exosomes can enhance sensitivity of cervical cancer to radiotherapy in vitro possibly by promoting apoptosis. In this case, forced expression of miR-22 via gene transfection results in inhibited expression of MYCBP and a subsequent decrease in hTERT, thereby promoting radiosensitivity of cervical cancer cells [96]. 

Melanoma is a melanocyte derived highly malignant tumor with strong invasiveness, high metastasis rate and rapid progression. Clinically, radiotherapy is the important treatment option for surgically unresectable melanoma. Studies have shown that exosomes derived from mesenchymal stem cells can improve the efficacy of radiotherapy in melanoma via bystander effect and distal effect, thus preventing the metastasis and spread of melanoma cells [97]. 

Breast cancer is one of the most common carcinomas endangering women’s health, and radiotherapy has become the standard treatment for locally advanced breast cancer. It has been shown that crosstalk between stromal cells and breast cancer cells acts through paracrine and juxtacrine signals, and exosomal RNAs regulate the radioresistance through anti-viral STAT1/NOTCH3 pathway [98]. Meanwhile, studies have found that exosomes released from breast cancer stem cells can induce autophagy, while autophagy has been proved to regulate the sensitivity of cancer to radiotherapy [89, 90]. Thomas et al. [99] demonstrated that proteomic characteristics of tumor-related exosomes may reflect the oxygenation state of breast cancer cells, indicating that it can be used for identifying irradiation resistant tumor cells. Moreover, it has been reported that ionizing radiation increases the activity of exosome secretory pathway in breast cancer cell line MCF-7, probably resulting in radioresistance in the cancer cells [79]. 

## Perspectives

**Fig. 5:**
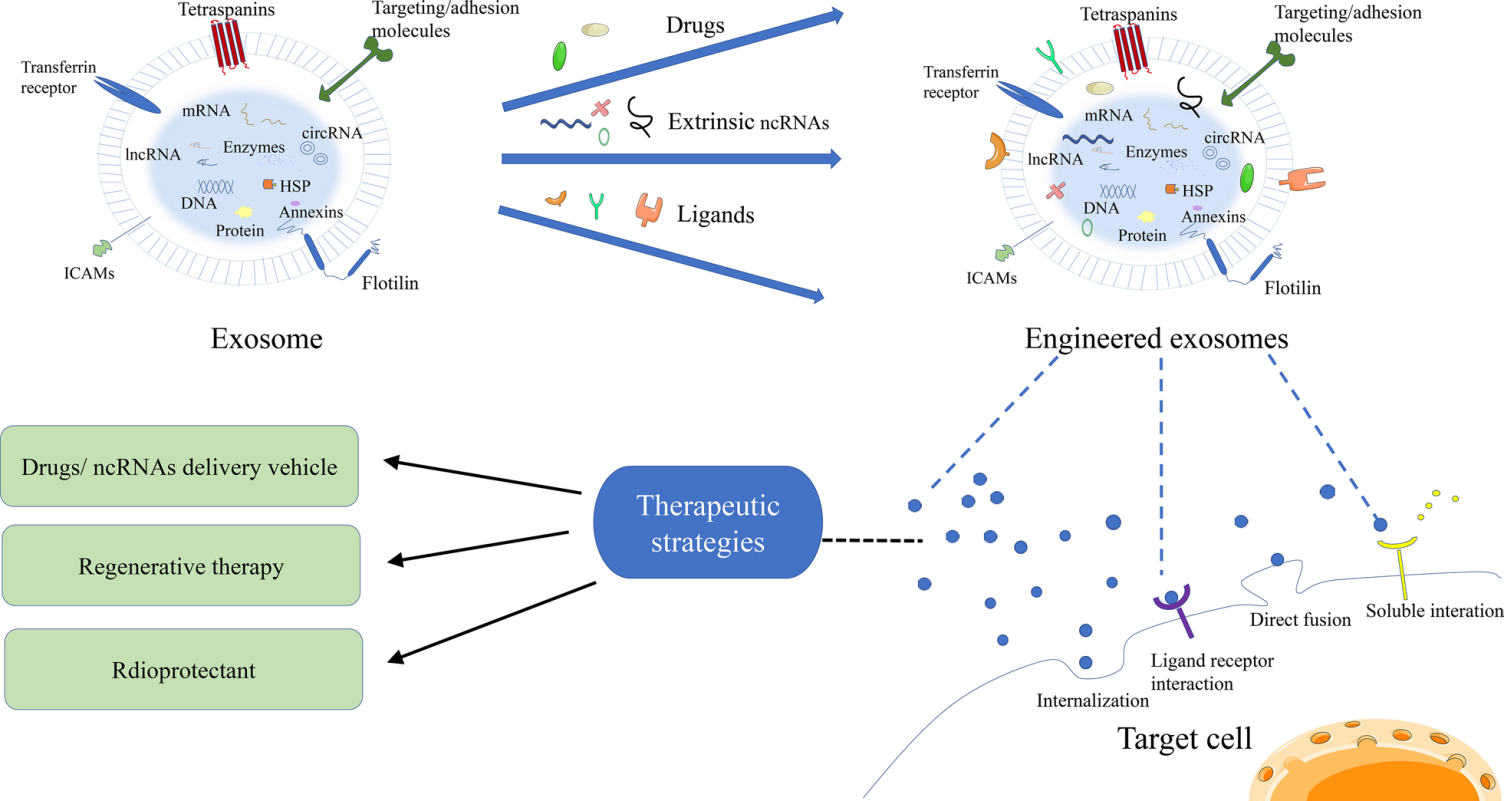
Clinical therapeutic strategies of exosomes in the field of cancer radiotherapy

Radiation therapy remains an important part of cancer treatment, and most cancer patients receive radiotherapy during their illness. Yet, tolerance to radiotherapy or even radioresistance is the main reason for radiation therapy failure, and there is an urgent need for biomarkers that can predict the efficacy of radiation therapy to screen the superior treatment population and assist in rapid clinical adjustment of treatment plans through real-time monitoring. Numerous studies have shown that exosomes can mediate resistance to radiotherapy, and changes in the expression levels of their contents are closely related to treatment response. Conversely, radiation can also affect the production, secretion, composition and uptake of exosomes [100]. These solid research foundations enable exosomes promising as biomarkers for monitoring radiosensitivity (Fig. 3). In addition to being a liquid biopsy tool, exosomes themselves as natural nanoscale vesicles are also a research hotspot in the field of cancer formation, progression, migration, invasion, metastasis and treatment. A growing number of studies have confirmed that exosomes can promote tumor development, thus, inhibition of exosome biosynthesis, secretion and uptake [101] has anti-tumor potential [101]. Based on this scenario, the following measures could be taken: blocking the generation and release of exosomes as well as exosome-mediated intercellular communications, e.g. decreasing exosome production using small-molecule enzymes and protein inhibitors; blocking adhesion molecules on the surface of exosomes such as phosphatidylserine, intercellular adhesion molecule 1 and its receptor in order to effectively decrease the uptake of exosomes; or disturbing the downstream signaling pathway in recipient cells elicited by exosomes. All the above strategies would provide new directions for cancer therapy. In the meantime, exosomes can be used as delivery systems for therapeutic drugs such as drugs and various nucleic acids such as mRNA/miRNA and other non-coding RNA/interfering small RNA (siRNA), and are expected to become an important tool for malignant tumor treatment through precision drug delivery. Besides, nano-scale diameter, lipid bilayer structure, high biocompatibility, high stability and low immunogenicity of exosomes enable exosomes to persist for a longer time in blood circulation, thus promoting tissue-directed transport as well as uptake of encapsulated contents of exosomes by the recipient cells. In these cases, while exosomes can selectively penetrate tumor tissues by enhancing permeability and retention effect due to their nano-scale diameter, the presence of phospholipid bilayer structure in exosomes protects their contents from bioenzyme-mediated hydrolysis for maintaining the activities of various biomolecules. In addition, the high biocompatibility allows exosomes to evade immune monitoring and penetrate into the tissue. Exosome-based drug delivery is currently undergoing extensive clinical trials, with preliminary evidence of safety of delivery and tolerability in oncology patients. Moreover, exosomes have been shown to be pivotal in the prevention and treatment of radiation-induced tissue injury [102–104]. As the risk of radiation exposure increases, it can damage normal tissue and cause various side effects. Traditional radioprotectants have limitations, therefore, it is essential to develop new radioprotectors, just in time exosomes as radioprotective agents hold great promise. In particular, MSC-derived exosomes showed tremendous advantages in the treatment of refractory graft vs. host disease (Fig. 5) [105]. Together, exosomes could become an extremely promising research direction in tumor radiotherapy.

To date, there have been content-rich research reports with novel entry points on exosomes at home and abroad. However, limited understanding of biogenesis, content sorting, subtype analysis, fluid transmission, target cell uptake, and content delivery of exosomes has greatly restricted accurate investigation on the role of exosomes in malignant tumor progression as well as individualized therapy [15]. The main challenges of exosome application in cancer therapy are as follows: how to effectively load exogenous ncRNAs or drugs into exosomes and further increase cell-specific delivery; how to prevent immune responses when utilizing non-autologous exosomes; how to prolong the half-life of modified exosomes in vivo, avoiding their rapid clearance; how to control the quality of exosomes administered to patients and the technical challenges associated with clinically graded production. Due to the complexity of exosome biology and these clinical challenges, it is critical to carefully establish standards for exosome quality and improve their effectiveness in vivo prior to their widespread use in clinical trials. Moreover, the mechanism underlying response of exosomes to ionizing radiation remains to be fully understood. Besides, the complete information of radiation-induced exosomes has yet to be fully unveiled. In short, there is still a long way to go for exosomes to achieve clinical translation, which requires the efforts of most researchers.

## Conclusion

The clinical application of exosomes in the field of radiation therapy for malignant tumors is still in the initial stage. There are several challenges facing radiotherapy specialists in the coming years, including elucidation of the mechanism underlying radiation-induced formation of exosomes, a fully understanding of impacts of the exosomes on the microenvironment and cell signaling transduction, reducing the probability of radioresistance and metastasis of tumor cells, and promoting the efficacy of malignant tumor radiotherapy. We expect exosomes will be used in clinical application as soon as possible with the continuous progress of technology, so that more tumor patients can benefit from them.

## Data Availability

The data supporting the conclusion of this review have been included within the article.

## Abbreviations

RIBE: Radiation-induced bystander effect
RIAE: Radiation-induced abscopal effects
AKT: Serine/threonine kinase 1
ATF2: Activating transcription factor 2
CRC: Colorectal cancer
EBV: Epstein-Barr virus
EC: Esophageal cancer
EEPD1: Endonuclease/exonuclease/phosphatase family domain containing 1
ESCC: Esophageal squamous cell carcinoma
FBXW7: F-box and WD repeat domain containing 7
FOXA1: Forkhead box A1
HNSCC: Head and neck squamous cell carcinoma
HPV: Human papilloma virus
HSP72: Heat shock protein family A (Hsp70) member 1A
IGF1R: Insulin like growth factor 1 receptor
KLF10: Kruppel-like factor 10
LMP1: Latent membrane protein 1
MAPK: Mitogen-activated protein kinase
NPC: Nasopharyngeal carcinoma
NSCLC: Non-small cell lung cancer
NOTCH2: Notch receptor 2
PBT: Proton beam therapy
PUM1: Pumilio RNA binding family member 1
TCEAL7: Transcription elongation factor A like 7
TGF-β: Transforming growth factor beta
TGFB1: Transforming growth factor beta 3
UVRAG: UV radiation resistance-associated gene
